# Benefits of a Specific and Supervised Rehabilitation Program in Femoroacetabular Impingement Patients Undergoing Hip Arthroscopy: A Randomized Control Trial

**DOI:** 10.3390/jcm10143125

**Published:** 2021-07-15

**Authors:** Alexis Müller-Torrente, Jordi Puig-Torregrosa, Sergio Montero-Navarro, Javier Sanz-Reig, Jaume Morera-Balaguer, Jesús Más-Martínez, Jesús Sánchez-Mas, Jose M. Botella-Rico

**Affiliations:** 1 Department of Physiotherapy, Alicante Clinic, 03010 Alicante, Spain; alexismuller1@yahoo.es; 2 Department of Traumatology, Traumadvance Clinic, 08222 Terrassa, Spain; jpuigt1797@gmail.com; 3 Department of Physiotherapy, Health Science Faculty, CEU-Cardenal Herrera University, CEU Universities, Plaza Reyes Católicos, 19, 03204 Elche, Spain; sergio.montero@uchceu.es (S.M.-N.); jmorera.el@uchceu.es (J.M.-B.); 4 Department of Orthopedic Surgery, HLA Clínica Vistahermosa, 03015 Alicante, Spain; javisanz@coma.es (J.S.-R.); jmas@traumavist.com (J.M.-M.); 5 Department of Biomedical Sciences, Health Sciences Faculty, CEU-Cardenal Herrera University, CEU Universities, Alfara del Patriarca, 46115 Valencia, Spain; jesus.sanchez@uchceu.es

**Keywords:** femoroacetabular impingement, hip arthroscopy, rehabilitation, physical therapy

## Abstract

(1) To assess the efficacy of a specific rehabilitation protocol for femoroacetabular impingement syndrome (FAIS), patients who underwent hip arthroscopy (HA) were compared with a control group. (2) Patients with symptomatic FAIS who were scheduled for HA were randomized either to a control group (*n* = 45, 66.6% men, 41.8 ± 12.4 years) following a general post-surgical treatment protocol or to an experimental group (*n* = 45, 71.2% men, 40.9 ± 7.6 years) following a specific rehabilitation protocol supervised by a physiotherapist. Range of motion (ROM), orthopedic tests and pain were assessed immediately before surgery and at 4 and 14 weeks after surgery. The hip functional status was assessed by the modified Harris Hip Score (mHHS) before surgery and at the end of follow-up. (3) At 14 weeks after surgery and compared with the control group, the experimental group showed a lower percentage of positives for hip provocation tests (15.6% vs. 46.6% on Faber test; 15.6% vs. 77.8% on Fadir test; 2.2% vs. 20% on Ober test, experimental vs. control group, *p* < 0.001), a greater improvement in mHHS (27.2 vs. 10.7 points, *p* < 0.001) and higher ROM for all the movements evaluated: flexion (99.6 ± 12.2 vs. 89.6 ± 4.5, *p* < 0.001), extension (20.6 ± 5.8 vs. 13.3 ± 2.6, *p* < 0.001), adduction (30.6 ± 5.7 vs. 23.4 ± 8.4, *p* < 0.001), abduction (43.4 ± 10.7 vs. 32.8 ± 8.4, *p* < 0.001) and both internal (28.2 ± 8.5 vs. 18.7 ± 6.1, *p* < 0.001) and external hip rotation (36.8 ± 9.3 vs. 27.4 ± 5.6. *p* < 0.001). The pain decreased after surgery for both groups, although the reduction was greater in the experimental group at the end of intervention (13.8 ± 16.1 vs. 34.9 ± 16.3 mm, experimental vs. control group, *p* < 0.001). (4) The specific and supervised rehabilitation program in patients with FAIS undergoing HA showed better benefits at 14 weeks of treatment than the benefits achieved by a care protocol in terms of pain reduction and recovery of hip motion.

## 1. Introduction

Femoroacetabular impingement syndrome (FAIS) is a well-known cause of hip pain in adolescents and young adults, with an overall incidence of FAIS diagnosis of 54.4 per 100,000 person-years [1]. There are two types; one of them is due to morphological abnormalities of the proximal femur, typically located on the anterolateral portion of the femoral head-neck junction, which is referred to as cam impingement, while acetabulum-related impingement is referred to as pincer-type [2,3] After forced flexion with an internal rotation, it can produce an abnormal contact between the femoral head-neck junction and the anterosuperior rim of the acetabulum, which over time can alter the function of joint sealing performed by the labrum. In some cases, it is thought that labral damage or combined chondrolabral pathology contributes to modify lubrication and the normal biomechanics of the hip joint [4,5]. This does not always happen, though FAIS morphology may increase the likelihood of hip pain and impaired performance exacerbated by physical activity, which occurs mainly in younger sporting populations who sustain repetitive flexion and rotational loading to their hip [6,7,8].

When non-operative management fails, hip arthroscopy (HA) is commonly used to recreate the spherical contour of the femoral head, improve femoral offset, normalize coverage of the acetabulum and repair/reconstruct chondral damage and the labrum to improve normal mechanics and joint sealing [9]. Being that it is a minimally invasive procedure with great benefits, its prescription has increased exponentially in the last ten years [10,11]. Although many studies exist that report post-HA rehabilitation, the vast majority of these studies lack specific detail related to the rehabilitation provided, and there is no current evidence-based consensus. In most cases, rehabilitation was based on the hip prosthesis guidelines, which resulted in a decrease in the effectiveness of rehabilitation [12]. Currently, more specific rehabilitation protocols have been described [13,14,15,16,17], but most of these have not shown data so far, and the few clinical outcomes described lack the high quality needed to support a specific protocol [17]. These protocols usually divide recovery into four stages focused on recovering muscle function and strength and improving joint range of motion (ROM) in order to facilitate a safe and graded return to sporting activity [16,18]. However, protocols can vary significantly with regard to postoperative restrictions, rehabilitation activities and time point for activities, and leave the selection of the treatment in each stage to the physiotherapist. Recent systematic reviews have noted the lack of comparative literature to guide postoperative rehabilitation and suggest the need for comparison trials toward a more specific rehabilitation following HA [14,15,17].

The main objective of this study was to design a specific and physical-therapist-supervised rehabilitation protocol for patients with FAI who underwent HA and compare it to the application of a standard post-surgical hip care protocol.

## 2. Materials and Methods

This study was approved by the ethics committee of the General University Hospital of Elche (PI 6/2019) and prospectively registered at clinicaltrials.gov (accessed on 14 July 2021) (NCT03959254). The study was carried out in compliance with the Good Clinical Practice and the Helsinki Declaration.

### 2.1. Participants

A single-blind study with a blinded evaluator strategy with two parallel groups was conducted. Patients with symptomatic cam FAIS (impingement-related hip or groin pain of greater than 3/10 on a visual analog scale for at least 3 months) and radiographic evidence of cam morphology (an alpha angle of 60° or greater on either anterior/posterior pelvic or Dunn 45° hip radiographs) who were scheduled for HA [19,20,21] were recruited from the surgical practices of two orthopedic surgeons in Alicante, Spain. Pincer and mixed types were excluded. All subjects followed the same surgical procedure: hip arthroscopic with an inside-out technique [22]. Exclusion criteria included: (1) having received physical therapy treatment in the past 3 months; (2) previous hip surgery or other major hip injury; (3) other musculoskeletal conditions including rheumatoid arthritis; (4) inability to perform testing procedures; (5) inability to attend a 12-week treatment program at baseline and follow-up assessments; (6) professional athlete; (7) radiographic evidence of hip osteoarthritis (more than Tönnis grade 2:3); (8) contraindications for the HA procedure; (9) other pathologies than can influence therapy effects, such as cardiovascular disease; (10) inability to speak or understand the Spanish language; (11) inability to comply with postoperative rehabilitation and exercises due to other reasons, such as a lack of time. All participants provided informed consent, and anonymity was ensured.

### 2.2. Interventions

**Figure 1:**
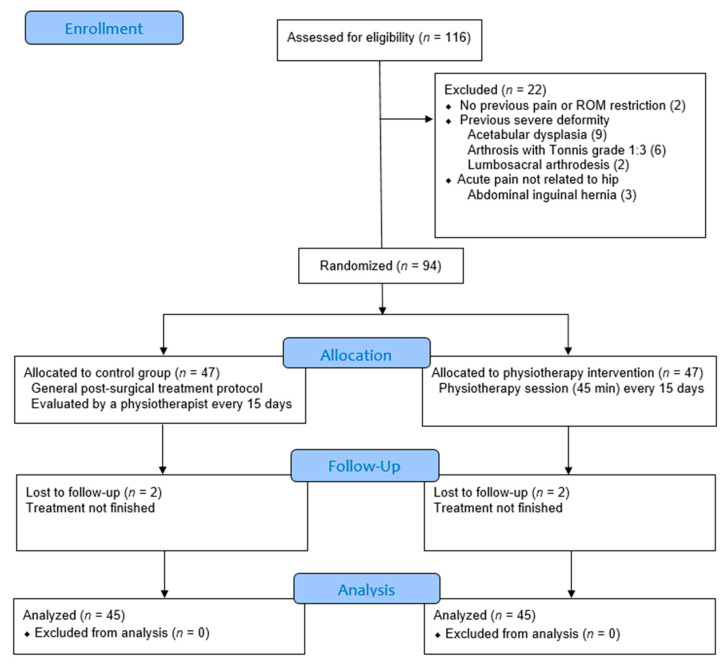
Flow diagram of the study protocol. Abbreviations: ROM (goniometry range of motion).

Eligible participants were randomized to an experimental group or a control group (ratio 1:1) on the day of the first post-surgery physiotherapy session by a staff member not involved in the trial and using numbered, nontransparent, sealed envelopes. All participants received adequate pre- and postoperative care, including health education and an exercise plan for the immediate postoperative period, in addition to a follow-up visit by the surgeon after two weeks of the intervention. The participants in the control group followed usual care. This protocol consists of an education program, including advice on movements that should be avoided, use of devices, posture, lifting and carrying, washing and bathing, nonspecific strengthening and stretching of lower limbs. The participants in the experimental group performed the same education program, early mobilization and walks, and in addition they followed a different program of exercises focused on stabilization, proprioception, flexibility and strengthening specifically designed to FAIS, following the guidance of the American College of Sports Medicine [23,24]. The program of exercises in the experimental group was applied in a physiotherapy session of 45 min each, once every two weeks for a total of 7 sessions (weeks 2, 4, 6, 8, 10, 12 and 14 post-surgery) following an adaptation of the Takla O’Donnell protocol [10], with the aim of restoring ROM and strength and reducing pain. This adaptation has been named the Müller and Puig protocol and consists of an education program and stabilization, proprioception, stretching and strengthening exercises adapted to the injuries produced by surgery and the deficiencies associated with the FAIS properly, to restore joint movement by protecting the capsule and cartilage in the early stages, progressing towards neuromuscular control with progressive muscle reeducation and strengthening exercises. The expanded protocols and the main differences between the control group and the experimental group are provided in the Supplementary Materials (Tables S1–S4). A flow chart outlining the study procedures is shown in Figure 1.

### 2.3. Outcomes

At inclusion, the authors collected information from participants about demographic data (gender, age), anthropometric data (weight, height) and physical activity (weekly). The volume of weekly physical activity was determined by asking the minutes per week that the participant performed a sports activity of moderate or vigorous intensity. ROM, orthopedic tests and pain were assessed immediately before surgery and at 4 and 14 weeks after surgery. The Visual Analogue Scale (VAS) was used to evaluate intensity of pain.

Hip motion was assessed by goniometry (ROM) and functional status by the modified Harris Hip Score (mHHS). Goniometric measurements were assessed by a physical therapist according to the method described by the American Academy of Orthopedic Surgeons (American Academy of Orthopedic Surgeons. Joint motion: Method of measuring and recording. Chicago: American Academy of Orthopedic Surgeons; 1965). The movements evaluated were flexion, extension, adduction, abduction and both internal and external rotation of the hip. The mHHS was used to evaluate the functional status of an individual and interference in the activities of daily life. The mHHS is a commonly used patient-reported outcome score used for the long-term evaluation of patients who had undergone hip arthroscopy, and it includes only assessments based on pain and function [25]. The mHHS is scored from 0 (worst functional outcome and maximum pain) to 100 points (best functional outcome and least pain). The values of the mHHS score are interpreted as follows: score < 70 points (poor result), 70–79 points (fair result), 80–89 points (good result) and 90–100 (excellent result).

To assess whether the pain persists in the area being evaluated, we used three orthopedic tests (Faber, Fadir and Ober Test) described in the Supplementary Materials.

The therapeutic adherence to the experimental protocol at 4 and 14 weeks after surgery was measured based on self-report of the subject using a specific diary, which monitors the performance and duration of exercises.

### 2.4. Statistical Analysis

Data were analyzed with SPSS version 22 (SPSS Inc., Chicago, IL, USA). Quantitative variables were expressed as means ± standard deviation. Proportions were used to describe statistical qualitative variables. The Kolmogorov-Smirnov test was used to determine whether the data of the variables followed a normal distribution or not. Unpaired t-tests or U Mann-Whitney tests were used for two-group comparisons, as appropriate. A one-way analysis of variance or Chi-square test was used for multiple comparisons, as appropriate. The Spearman’s correlation was used to assess associations between variables. Cohen’s kappa was used to measure interrater reliability. Statistical significance was assumed at *p* < 0.05.

## 3. Results

A total of 116 participants were assessed for eligibility, 22 of whom were excluded (Figure 1).

Eligible participants (*n* = 94) were randomized to an experimental group or a control group on the day of the first post-surgery physiotherapy session, but two participants from each group were excluded at follow-up because they did not finish the treatment. Anthropometric parameters and physical activity of the respective groups are shown in Table 1.

The total population was mostly male (68.9%), with a mean age of 41.3 ± 10.2 years, a body mass index (BMI) of 24.3 ± 3.7 kg/m^2^ and a physical exercise practice of 5.0 ± 3.1 h/week. There were no significant differences between the control and experimental group for any of these parameters.

Pain persistence was checked immediately before surgery and at 4 and 14 weeks after surgery through the performance of three orthopedic tests (Table 2).

Both the control and experimental group showed a reduction in the percentage of positives for the three tests evaluated at 14 weeks post-surgery, but not at 4 weeks post-surgery. The improvement was significantly greater in the experimental group, which reached at 14 weeks post-surgery a lower percentage of positives than the control group for the Faber test (15.6% vs. 46.6%, experimental vs. control), Fadir test (15.6% vs. 77.8%) and Ober test (2.2% vs. 20%). ROM was also assessed (Table 3).

**Figure 2:**
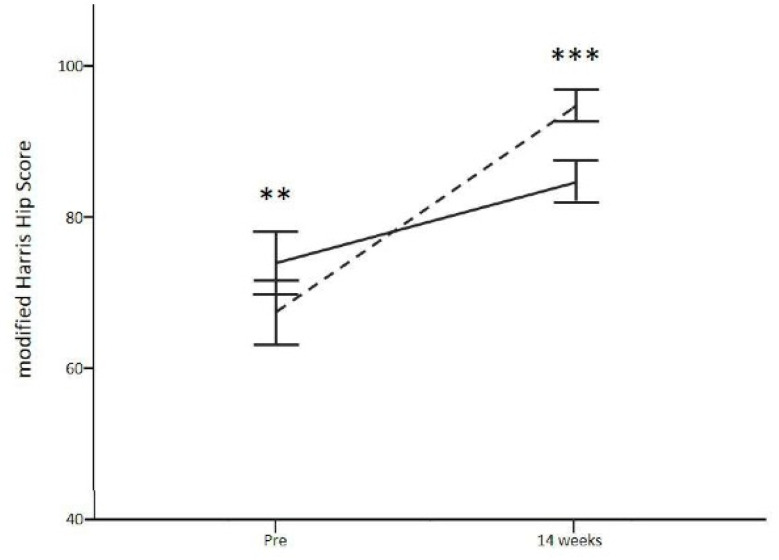
Functional status and interference in the activities of daily life were evaluated by mHHS. ** *p* < 0.01; *** *p* < 0.001 vs. control group.

All the movements evaluated (flexion, extension, adduction, abduction and both internal and external rotation) showed a significant improvement at 14 weeks post-surgery compared with the pre-surgery score for the experimental group. Regarding the control group, extension and abduction movements showed no significant improvement at follow-up. When comparing between groups, the improvement shown at 14 weeks after surgery was significantly higher for the experimental group compared to the control group for all movements evaluated: flexion (99.6 ± 12.2 vs. 89.6 ± 4.5, *p* < 0.001), extension (20.6 ± 5.8 vs. 13.3 ± 2.6, *p* < 0.001), adduction (30.6 ± 5.7 vs. 23.4 ± 8.4, *p* < 0.001), abduction (43.4 ± 10.7 vs. 32.8 ± 8.4, *p* < 0.001) and both internal (28.2 ± 8.5 vs. 18.7 ± 6.1, *p* < 0.001) and external rotation (36.8 ± 9.3 vs. 27.4 ± 5.6 *p* < 0.001). The pain, assessed by VAS, decreased progressively after surgery for both groups. When comparing between groups, the experimental group showed a significantly greater reduction in pain at 14 weeks after surgery than was observed in the control group (13.8 ± 16.1 vs. 34.9 ± 16.3 mm, experimental vs. control group, *p* < 0.001). The greatest decrease in perceived pain and better functional recovery for the experimental group at the end of the intervention compared to the control group was confirmed with mHHS (Figure 2).

Experimental and control groups had a pre-surgery mHHS of 67.2 ± 14.3 (poor result) and 73.7 ± 13.9 (fair result), respectively, with significant differences between groups (*p* = 0.003). At 14 weeks after surgery, the mHHS of the experimental and control group was 94.4 ± 7.1 (excellent result) and 84.4 ± 9.4 (good result), respectively (*p* < 0.001). The improvement showed in the experimental group at 14 weeks post-HA was 27.2 points, significantly greater than that obtained in the control group (10.7 points). Finally, the adherence to the Müller and Puig protocol reported by the experimental group at the end of the intervention was 91.5 ± 5.6%. It should be noted that those patients with greater adherence to treatment reported lower pain perception evaluated with VAS (r = −0.544, *p* < 0.001), as well as a higher score in mHHS (r = 0.426, *p* = 0.004).

## 4. Discussion

The main findings are summarized as: (i) this study has allowed us to evaluate the effect of the inclusion of a specific treatment supervised by a physiotherapist in the rehabilitation of FAIS post-HA; (ii) the specific treatment improved functional recovery and interference in the activities of daily life, and decreased pain, with respect to the control group; (iii) the beneficial effect was greater in those patients who showed more adherence to the treatment supervised by the physiotherapist. These findings support the addition of a specific physical therapy supervised by a professional to usual care for patients with FAIS who have undergone HA.

Currently, bibliographic reviews demonstrate that there is a lack of high-quality literature that suggests an effective protocol for postoperative rehabilitation of HA for FAIS [15,17,26]. Thus, the description of postoperative rehabilitation programs is lacking or poorly reported in most of the literature, and further comparative trials to determine the effect of specific postoperative rehabilitation designs are suggested [14,27]. In this sense, one of the strengths of our study is the existence of a control group. Most post-HA rehabilitation studies in patients with FAIS have shown results from their protocol without comparing to a control group. From our knowledge, there is only one previous study that has evaluated the efficacy of adding a physiotherapist-prescribed rehabilitation program to arthroscopic surgery for FAIS [12]. Thus, Bennell et al. showed significantly greater improvements on the Copenhagen Hip and Groin Outcome Score (HAGOS), the International Hip Outcome Tool (iHOT-33) and the sport subscale of the Hip Outcome Score (HOS) for the physiotherapy group at week 14 post-HA. However, the results were considered preliminary due to the small sample size (14 per group). Similarly, our study proposes a specific rehabilitation protocol with follow-up at 4- and 14-weeks post-surgery whose objective is to normalize ROM, strength and pain, but performed on a larger sample and assessing different variables.

The first aspect to evaluate was the persistence of pain through orthopedic tests. After 4 weeks post-HA, the pain remained persistent for the majority of the participants without significant changes observed with respect to the evaluation prior to surgery, both for the control group and for the experimental group. At 14 weeks, the Ober test improved in the control group, although it was less evident in the Faber and Fadir tests. These results are in agreement with the study by Palmer et al. [28], where at 8 months post-HA, the usual care showed no significant improvement for the Fadir test compared to pre-surgery, and the change shown in the Faber test was similar to that reached in our study. However, the Müller and Puig protocol was able to reduce the percentage of positives for the three orthopedic tests evaluated with respect to the control group at 14 weeks post-surgery, reducing the presence of pain in the Fadir and Faber tests below 20%, and up to only 2% in the Ober test. These results show that a specific physiotherapy supervised by physiotherapists has a greater benefit than usual care, both in our study and in data published by other authors [28]. The Müller and Puig program achieved better results in the Fadir test at 14 weeks, probably due to the adaptation of the exercises for the protection of the structures involved in arthroscopy (acetabulum, labrum, femur and capsule). The protection of these structures during the healing process suggests a higher quality of tissue in the coxofemoral and less irritability to compression (Fadir test) once the process is finished. The rest of the tests (Faber and Ober), being extensibility tests, obtained similar improvements to those of the control, since the exercises in this respect were similar between the study groups. Probably, the main point of improvement of the Müller and Puig protocol lies in the protection of the structures affected by the surgery and the pathophysiology of the FAIS, which allows for more effective healing and with it a faster and greater recovery than protocols that do not take into account the pathological peculiarities of FAIS and its arthroscopic surgery.

Regarding hip motion, a significant increase in ROM was observed for each of the movements analyzed in the experimental group with respect to the control group at 14 weeks after surgery, but not at 4 weeks post-surgery. Regarding hip motion, a significant increase in ROM for flexion, adduction and internal or external rotation of the hip was observed at 14 weeks after surgery, both for the control and experimental groups, but not at 4 weeks after surgery. Extension and abduction only improved significantly in the experimental group, and in any case, the improvement was greater in the experimental group compared to the control group for all movements analyzed. These results are in agreement with previous studies where postoperative physiotherapy produced a significant 9° improvement in hip flexion between 6 and 8 months post-HA [28,29], but it was not observed at 2 weeks post-HA [29]. It should be noted that, in our study, the group subjected to the experimental protocol showed a 15° increase in hip flexion at 14 weeks. In addition, significant improvement was obtained for extension, abduction, adduction and internal and external hip rotation, unlike the study by Palmer et al. [18] that did not observe significant recovery for these movements.

In addition, regarding recovery of the ROM of the hip, a recovery of functional status evaluated through the mHHS was observed at 14-weeks post-surgery for both the control group and for the experimental group. The control group showed 73.7 points as the pre-surgery score (fair result), while the experimental group showed 67.2 points (poor result). However, at 14 weeks after surgery, the mHHS of the experimental group achieved 94.4 points (excellent result), better than that achieved by the control group with 84.4 points (good result). These results are in agreement with other previous studies, where post-HA physiotherapy rehabilitation improved mHHS [30,31,32,33,34]. However, the rehabilitation performed in these previous studies did not achieve the improvement obtained by our experimental protocol, which was 27.2 points at 14 weeks post-HA. In this sense, Spencer-Gardner et al. [33] reported a mHHS value of 80.1 in patients undergoing hip arthroscopy and a subsequent five-phase rehabilitation protocol with a 1-year follow-up, a value similar to that reached in our control group (84.4), but lower than that shown in the experimental group (94.4). Thorborg et al. [34] obtained an improvement of 17.1 points at 3 months after surgery, reaching 22 points at 12 months, lower than the improvement obtained in our study at 14 weeks. Similar results were obtained by Avnieli et al. [30] comparing postoperative weightbearing protocols with a 2-year follow-up (improvement was lower than 22 points). Only the study by Frank et al. [32] achieved a greater improvement (30.4 points) in cyclists undergoing HA for FAIS but was reported between 24 and 48 months post-surgery. In any case, this progressive improvement associated with the duration of the intervention suggests the need for a longer follow-up that permits the evaluation of the Müller and Puig protocol effect in the long term.

Finally, both the improvement observed in orthopedic tests and in mHHS showed a reduction in perceived pain after the application of the Müller and Puig protocol. Pain reduction is a common improvement in the rehabilitation of patients with FAIS undergoing hip arthroscopy in both the short and medium term after surgery [13,28,29,32,34,35,36]. In our study, this reduction in perceived pain was also confirmed by VAS. The experimental group showed a reduction in pain that was significantly greater than that observed in the control group. This decrease in pain observed in the group undergoing the Müller and Puig program was progressive, going from 68 mm before surgery to 46.9 mm after 4 weeks of rehabilitation, and decreasing to 13.8 mm after 14 weeks of rehabilitation. These results are consistent with the short-term effect of rehabilitation on pain evaluated by Cunningham et al. [29], in which patients with FAIS who underwent HA showed a reduction in pain from the first day after surgery with favorable evolution evaluated until 6 weeks post-surgery. Notably, the pain reduction evaluated by mHHS and VAS was greater in those patients who showed greater adherence to the Müller and Puig rehabilitation program, which confirms the benefits of using a specific and supervised rehabilitation program.

### Study Limitations

The study was carried out in the medium and short term, showing improvement at 14 weeks post-surgery. Although this improvement turned out to be higher and earlier in terms of recovery of hip functional status and reduction of pain than what was observed in our control group and in other previous studies, it is necessary to carry out a long-term follow-up to know if this improvement continues to progress over time. Furthermore, it is necessary to evaluate in new studies if the benefits observed with mHHS are reproduced in other scales used in the evaluation of hip functional status, such as iHOT33, HOS and HAGOS, which cover different dimensions. This study attempts to demonstrate the efficacy of a postoperative hip treatment adapted to the characteristics of the FAIS. When compared with a generic protocol for hip rehabilitation, we verified the importance of adapting the exercises to the characteristics of the FAIS and its surgery, abandoning the generalized practice of applying nonspecific protocols to postoperative hip treatments. However, it would be necessary to compare the effectiveness of our protocol with other specific programs for FAIS.

Finally, orthopedic tests (Faber, Fadir and Ober test) were used in the study as an outcome measure, although they are validated as diagnostic tests. The use of these tests tries to reinforce the benefits of the program by limiting the occurrence of pain in positions of maximum combined mobility of the hip. We tried to prioritize ROM deficits over orthopedic test results. The results obtained suggest that the correlation between the positivity of these tests and the specific loss of ROM could be assessed in the future as a diagnostic or outcome measure [37].

## 5. Conclusions

In summary, the rehabilitation program proposed in this study, and supervised by a physiotherapist, has been shown to achieve better benefits than the usual care in terms of pain reduction and recovery of hip motion. These results suggest adapting usual care in order to improve the recovery of the patient with FAIS undergoing HA with an inside-out technique and proposing the Müller and Puig protocol as a model to consider.

In summary, the rehabilitation program proposed in this study, and supervised by a physiotherapist, has been shown to achieve benefits in terms of pain reduction and recovery of hip mobility in patients with FAIS undergoing HA with an inside-out technique. Considering the limitations of this study, the results suggest that the protocol proposed by the authors could be a model to consider for the rehabilitation of these patients.

## Figures and Tables

**Table 1:** Characteristics of the studied population.

	Total (*n* = 90)	Control *(n* = 45)	Experimental (*n* = 45)	*p*
Population (%)	100	50	50	
Sex (% men)	68.9	66.6	71.2	
Age (years)	41.3 ± 10.2	41.8 ± 12.4	40.9 ± 7.6	0.676
Height (cm)	173 ± 9.6	172 ± 11.2	175 ± 7.4	0.060
Weight (kg)	73.5 ± 14.3	73.8 ± 15.0	73.3 ± 13.6	0.855
BMI (kg/m^2^)	24.3 ± 3.7	25.0 ± 3.9	23.7 ± 3.4	0.140
Physical activity (h/week)	5.0 ± 3.1	5.2 ± 2.3	4.8 ± 3.8	0.547

Data shown as mean ± standard deviation or percentage (%). BMI, body mass index. *p* values do not indicate significant differences between control and experimental groups.

**Table 2:** Analysis of orthopedic tests.

Test		Pre	4 Weeks	14 Weeks	χ^2^	*p*
FABER	Control	57.8 (26)	71.2 (32)	46.6 (21)	5.554	0.062
	Experimental	95.6 (43)	97.8 (44)	15.6 (7)	93.386	*p* < 0.001
FADIR	Control	88.8 (40)	95.6 (43)	77.8 (35)	6.595	0.037
	Experimental	93.4 (42)	84.4 (38)	15.6 (7)	71.185	*p* < 0.001
OBER	Control	46.6 (21)	66.6 (30)	20.0 (9)	19.980	*p* < 0.001
	Experimental	51.2 (23)	44.4 (20)	2.2 (1)	28.794	*p* < 0.001

Data shown as percentage (%) of positive tests (n). Pre, before surgery; 4 weeks, 4 weeks after surgery; 14 weeks, 14 weeks after surgery; χ^2^**,** Pearson’s Chi-square value; *p*, significance resulted from Chi-square contingency test; FABER, passive flexion-abduction-external rotation test; FADIR, Passive flexion, adduction and internal rotation test; Ober, Ober test.

**Table 3:** Hip ROM and pain assessment.

	Control Group		Experimental Group			*p*
		differences		differences	differences	
		within groups		within groups	between groups	
Flexion		vs Pre-HA		vs Pre-HA	experimental vs. control	
Pre-HA	85.5 ± 9.3	-	84.6 ± 14.3	-	−0.9°	0.750
4 weeks	84.9 ± 4.9	−0.6°	84.6 ± 5.8	0°	−0.3°	0.918
14 weeks	89.6 ± 4.5 ^b^	+4.1°	99.6 ± 12.2 ^b^	+15.2°	+10.0°	<0.001
Extension						
Pre-HA	13.5 ± 3.2	-	14.9 ± 3.7	-	+1.4°	0.086
4 weeks	12.7 ± 2.3	−0.8°	13.4 ± 2.7 ^b^	−1.5°	+0.7°	0.212
14 weeks	13.3 ± 2.6	−0.2°	20.6 ± 5.8 ^b^	+5.7°	+7.3°	<0.001
Abduction						
Pre-HA	30.1 ± 10.1	-	33.8 ± 10.7	-	+3.7°	0.035
4 weeks	29.3 ± 7.5	−0.8°	30.7 ± 9.6	−3.1°	+1.4°	0.311
14 weeks	32.8 ± 8.4	+2.7°	43.4 ± 10.7 ^b^	+9.6°	+10.6°	<0.001
Adduction						
Pre-HA	20.6 ± 5.7	-	21.5 ± 6.9	-	+0.9°	0.474
4 weeks	21.2 ± 4.0	+0.6°	20.5 ± 5.8	−1.0°	−0.7°	0.344
14 weeks	23.4 ± 8.4 ^b^	+2.8°	30.6 ± 5.7 ^b^	+9.1°	+7.2°	<0.001
Internal rotation						
Pre-HA	16.6 ± 5.9	-	17.1 ± 7.5	-	+0.5°	0.855
4 weeks	16.8 ± 3.7	+0.2°	16.9 ± 5.4	−0.2°	+0.1°	0.670
14 weeks	18.7 ± 6.1 ^b^	+2.1°	28.2 ± 8.5 ^b^	+11.1°	+9.5°	<0.001
External rotation						
Pre-HA	24.9 ± 6.9	-	27.1 ± 9.3	-	+2.2°	0.216
4 weeks	24.7 ± 4.0	−0.2°	24.9 ± 7.6	−2.2°	+0.2°	0.743
14 weeks	27.4 ± 5.6 ^b^	+2.5°	36.8 ± 9.3 ^b^	+9.7°	+9.4°	<0.001
VAS (mm)						
Pre-HA	56.0 ± 22.6	-	68.0 ± 21.2	-	+12 mm	0.011
4 weeks	49.6 ± 16.6	−6.4 mm	46.9 ± 13.3 ^b^	−21.1 mm	−2.7 mm	0.391
14 weeks	34.9 ± 16.3 ^b^	−21.1 mm	13.8 ± 16.1 ^b^	−54.2 mm	−21.1 mm	<0.001

Data shown as mean ± standard deviation. Pre, before surgery; 4 weeks, 4 weeks after surgery; 14 weeks, 14 weeks after surgery. *p* values in bold indicate statistically significant differences between groups. ^b^, significantly different compared with baseline score within same group (*p* < 0.05). mm, millimeters; ROM, range of motion; VAS, visual analogue scale; Pre-HA, Pre hip arthroscopy.

## Supplementary Materials

The following are available online at https://www.mdpi.com/article/10.3390/jcm10143125/s1, Table S1: Sequence of exercises and weeks when practiced. Table S2: Grouping of exercises by category and description. Table S3: Differences between the control group and the experimental group.
