# Supplementary material for: Benefits of a Specific and Supervised Rehabilitation Program in Femoroacetabular Impingement Patients Undergoing Hip Arthroscopy: A Randomized Control Trial
Source: J Clin Med. 2021 Jul 15;10(14):3125. doi: 10.3390/jcm10143125 (PMC8307353; doi:10.3390/jcm10143125)
Supplement: Supplementary file 1 [file jcm-10-03125-s001.zip › jcm-1243888-supplementary.pdf]

SUPPLEMENTARY MATERIAL

The Müller & Puig protocol differs from the usual care in the following aspects: a) avoiding the tension of the capsulotomy and the iliofemoral ligament by avoiding external rotation and anterior hip translation; b) protecting the repair of labrum, acetabular cartilage and bone by avoiding critical movements, delaying the beginning of cycle exercises and focusing the exercises first on extension range of mobility, then on abduction and adduction; c) returning to activity by limiting the activity of the psoas, due to its anatomical relationship with the capsule and the anterior labial area. These adaptations have allowed the development of a more specific protocol for the rehabilitation of patients with FAI who have undergone HA with significant improvements over the usual care protocol. Patients were also instructed to perform early mobilization, functional exercises and nonspecific stretching and strengthening that included ankle movements, quadriceps and gluteal activation, active hip flexion and abduction, and daily walks, gradually increasing the distance. Therapeutic adherence to the Müller & Puig protocol was measured based on self-reporting of the participant using a specific diary which monitored the performance and duration of exercises.

Outcomes

Orthopedic tests used in this study:

- Faber test. The patient's hip was flexed, abducted and externally rotated by placing the external malleolus on the knee of the contralateral leg. The pelvis was stabilized, and an overpressure was applied to the inside of the knee. It was positive if the pain in the buttock or groin was reproduced. Test with inter-evaluator reliability in Kappa values (95% CI) of 0.60.
- Fadir test. Passive flexion, adduction and internal rotation test. With the patient supine, the evaluator patiently brought the patient's hip up to 100º flexion and adduction while applying

internal rotation. It was positive if pain in the groin was reproduced. Test with inter-evaluator reliability in Kappa values of 0.48.

- Ober test. Patient in lateral decubitus position. The evaluator flexed the knee at 90º and abducted and extended the hip until it was level with the trunk. The evaluator let gravity bring the hip on adduction to the extent possible. It was positive if the hip failed to adduce. Test with inter-evaluator reliability of 0.90.

1. **Müller&Puig protocol**

A. Stages and aims.

STAGE I: Weeks 1-4
Aims:
Protect integrity of repaired tissue
• Restore ROM within restrictions
• Diminish pain and inflammation
• Prevent muscular inhibition
Caution: loads should be limited. Do not force hip flexion or rotation.
Progression to stage 2: Pain below 20 on VAS when performing exercises, ROM greater than 75% on the healthy side, full load authorized.

STAGE II: Weeks 5-8
Aims
• Protecting integrity of repaired tissue
• Restoring full ROM
• Restoring normal gait pattern
• Progressively increasing muscle strength
Criteria for progression to Stage III
• Full range of motion
• Pain-free/normal gait pattern
All the series of exercises with the resistance prescribed may be carried out when pain is below 20 on VAS and provided there is no volitional muscular failure.

STAGE III. Weeks 9-14
Goals
• Restoring muscle endurance/strength
• Restoring cardiovascular endurance
• Optimizing neuromuscular control/balance/proprioception

B. Temporary execution of exercises.

Table S1. Sequence of exercises and weeks when practiced.

WEEKS	1	2	3	4	5-6	7-8	9-11	12-14
**1. CIRCULATION EXERCISES AND CONTRACTURE PREVENTION**								
1.1. Active ankle movements	X	X	X	X				
1.2. Quadriceps isometric	X	X	X					
1.3. Isometric of gluteus maximus	X	X	X					
1.4. Keep prone position	X	X	X					
1.5. Keep sitting position	X	X	X					
1.6. Keep semi-lying position	X	X	X					
**2. RESTORING THE RANGE OF MOTION**								
2.1. Active hip rotations	X	X						
2.2. Active hip flexion	X	X	X					
2.3. Seated trunk flexion			X	X	X			
2.4. Manual Therapy: Global Hip Mobilization		X	X	X	X	X		
2.5. Aquatic exercise: Assisted hip abduction and adduction				X	X			
2.6. Aquatic exercise: Active hip flexion-extension				X	X			
2.7. Aquatic exercise: Buoyancy assisted flexion			X	X				
2.8. Aquatic exercise: Cycling movement				X	X			
2.9. Quadriceps stretching						X	X	X
2.10. Hamstring stretching						X	X	X
2.11. Adductor stretching						X	X	X
2.12. Pyramidal stretching						X	X	X
**3. MUSCLE STRENGTHENING**								
3.1. Prone active hip extension with knee extension		X						
3.2. Prone active hip extension with knee in semiflexion		X						
3.3. Prone active hip extension with knee flexion		X						
3.4. Standing active hip abduction			X	X				
3.5. Side-lying active hip abduction				X	X	X		
3.6. Active knee extension		X	X					
3.7. Gluteal bridge			X	X				
3.8. Gluteal bridge with adductor activation			X	X	X			
3.9. Prone resisted hip extension with knee extension			X	X	X			
3.10. Prone resisted hip extension with knee in semiflexion			X	X	X			

3.11. Prone resisted hip extension with knee flexion			X	X	X			
3.12. Side-lying resisted hip abduction					X	X	X	
3.13. Standing resisted hip extension with knee extension						X	X	X
3.14. Standing resisted extension with knee in semiflexion						X	X	X
3.15. Standing resisted extension with knee flexion						X	X	X
3.16. Resisted knee extension			X	X	X	X		
3.17. Isometric hamstring exercise with Swiss Ball					X	X	X	
3.18. Swiss Ball wall squat					X	X	X	X
**4. NEUROMUSCULAR CONTROL**								
4.1. Load transfer					X			
4.2. Monopodal stance with knee flexed.					X	X		
4.3. Proprioception on unstable base						X	X	
4.4. Proprioception on an unstable base with active hip movement						X	X	
4.5. Proprioception on an unstable base with elastic band.							X	X
4.6. Gluteal bridge with unilateral support						X	X	
4.7. Walking heel to toe in water				X	X	X		
**5. CARDIOVASCULAR TRAINING**								
5.1. Stationary bicycle				X	X	X	X	X
5.2. Elliptical movement						X	X	X
**6. TRUNK EXERCISES**								
6.1. Side-lying isometrics using knees						X	X	X
6.2. Lying-face-down isometrics using knees						X	X	X
6.3. Exercise ball sit-ups						X	X	X
6.4. Quadruped extension					X	X	X	X

C. Description of the exercises

Load magnitude: maximum load withstood by the subject when completing all the repetitions in each set.
Repetitions: 10 to 20
Sets: 1 to 4
Rest between repetitions: 1 to 3 seconds (s)
Rest between sets: 30 to 90 s
Volitional muscular failure: No
Load progression. The magnitude of the load is increased subject to two conditions: a) The Visual Analogue Scale (VAS) after the exercise is less than 2; b) 10 repetitions are completed without volitional muscular failure.

Table S2. Grouping of exercises by category and description.

EXERCISE	DESCRIPTION	INTENSITY AND FRECUENCY	ILLUSTRATION
**1. CIRCULATION EXERCISES AND MUSCLE CONTRACTURE PREVENTION**			
1.1. Active ankle movements	The participant in a supine position, head resting on the treatment table. The lower limb resting on the table except for the heel extended beyond the edge. The participant alternates active flexion-extension and circumduction movements, reaching ROM limits on both sides.	**Sets**: 4<br>**Repetitions**: 10<br>**Sequence**:<br>3 s flexion,<br>3 s extension,<br>6 s full circumduction<br>**Rest between repetitions:** 3 s.<br>Twice a day	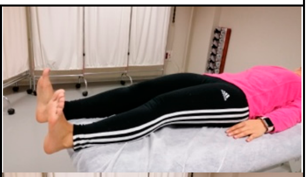<br>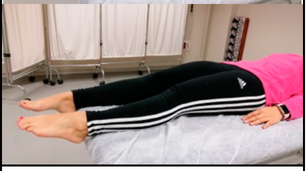
1.2 Isometric quadriceps.	The participant in a supine position, head resting on the table. A small ball (10 cm diameter) is placed between the table and the knee, which is in nearly full extension. The participant then squeezes the knee against the ball by isometric quadriceps contraction, then returning to the starting position.	**Sets**: 3<br>**Repetitions**: 10<br>6 s per contraction.<br>**Rest between repetitions**: 3 s.<br>**Frequency**:<br>Twice a day	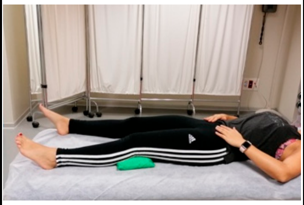
1.3. Isometric gluteus maximus exercise	The participant in a supine position, head resting on the table. The subject should attempt to raise the pelvis without lifting the body away from the table.	Same as exercise 1.2.	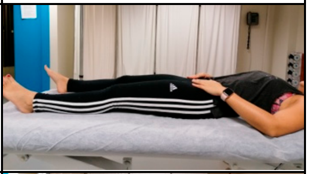
1.4. Sustained prone position	The participant holds a prone position, head resting to one side. If no tension is perceived around the hip flexors, a pillow may be placed between table and thigh.	**Time**: 15 minutes<br>**Frequency**: 4 times a day	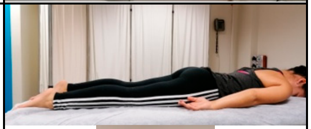<br>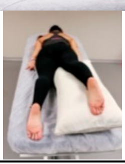

1.5. Sustained sitting position	The subject remains in a sitting position with 90º of hip flexion and feet resting on the floor. Arms should rest on the chair with the lower back supported by the back of the chair.	Same as exercise 1.4.	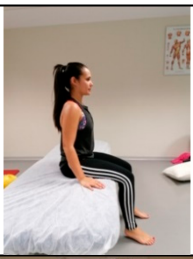
1.6. Sustained semi-lying position	The subject remains lying with the trunk slightly raised in a position between supine and sitting.	Same as exercise 1.4.	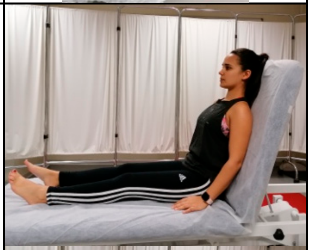 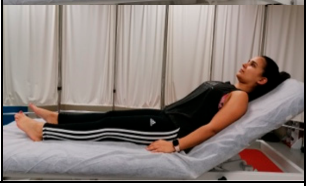
**2. RESTORING RANGE OF MOTION**			
2.1. Active hip rotations	The participant in a supine position, with the knee in extension. Subject mobilizes the hip in rotation by moving the tip of the foot medially and laterally in a pain-free range of motion, while the lower limb remains resting on the table..	**Sets**: 2<br>**Repetitions**: 10<br>**Sequence**:<br>3 s internal rotation,<br>3 s external rotation.<br>**Rest between repetitions**: 3 s<br>**Frequency**:<br>Twice a day	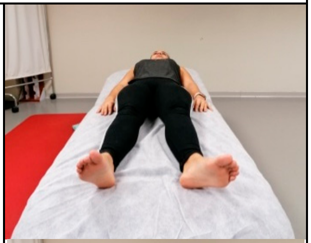 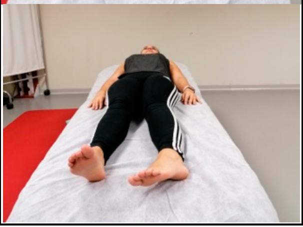

2.2. Active hip flexion	The participant in a supine position, with the head and the lower limb resting on the table. The subject bends hip and knee by sliding the heel along the table. The participant progresses from knee extension to maximum free hip flexion, lifting the heel if necessary in the last degrees of hip flexion. Then slowly return to the starting position.	**Sets**: 4<br>**Repetitions**: 10<br>**Sequence**: concentric 5 s, eccentric 5 s.<br>**Rest between repetitions**: 2 s.<br>**Frequency**: Twice a day	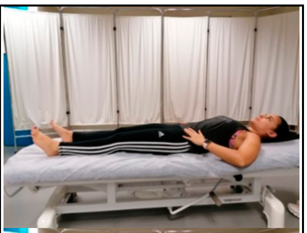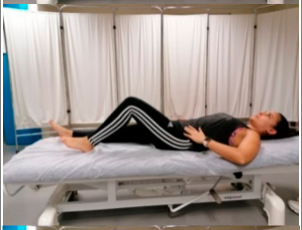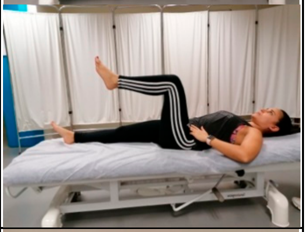
2.3. Seated trunk flexion	The participant is sitting in a chair with feet flat on the floor. The participant should bend forward to touch the feet without feeling any pain. The amplitude of the movement will be determined by the appearance of pain.	**Sets**: 2<br>**Repetitions**: 10<br>**Sequence**:<br>3 s trunk flexion;<br>1 s hold position;<br>3 s return to neutral trunk position.<br>**Rest between repetitions**: 3 s<br>**Frequency**: Once a day	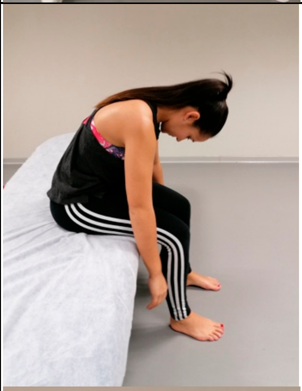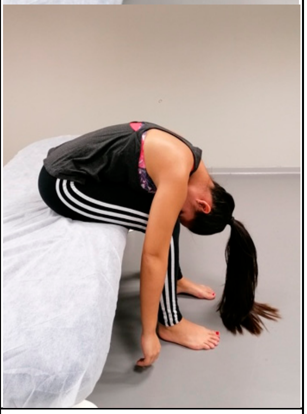
2.4. Manual Therapy: Global Hip Mobilization	The subject is lying supine with feet on the couch. The therapist performs joint mobilization with combined flexion-extension and abdominal-adduction movements, avoiding wide rotations. No pain should appear. The amplitude of motion is d and terminated by the onset of pain.	**Sets:** Two 5-minute sets.<br>**Frequency**: Once a day	

2.5. Aquatic exercise: Assisted hip abduction and adduction	Provided injury has properly healed, the participant stands in a pool with the water at chest level. The subject moves slowly sideways.	**Sets:** 3<br>**Repetitions:** 10<br>**Rest between series:** 30 s	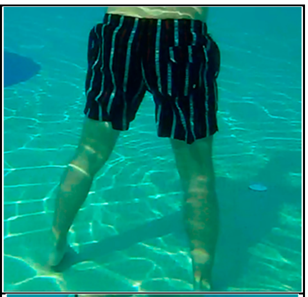
2.6. Aquatic exercise: Active hip flexion-extension	Provided injury has properly healed, the subject stands in a pool with the water at chest level. The subject slowly performs hip flexion and extension movements with the knee in extension.	**Sets:** 3<br>**Repetitions:** 10<br>**Rest between series:** 30 s	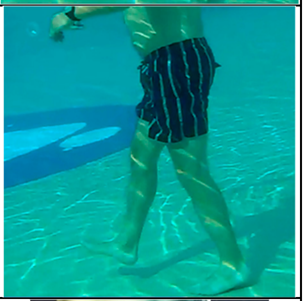
2.7. Aquatic exercise: Buoyancy assisted flexion	Provided injury has properly healed, the participant stands with the water at chest level. The subject places a tubular floater under the flexed knee of the affected side. The buoyancy resistance assists the hip flexion movement. Return to the initial position by sinking the floater with our hands.	**Sets:** 3<br>**Repetitions**: 10. Maintain flexion 6 s in each repetition.<br>**Rest between series:** 30 s	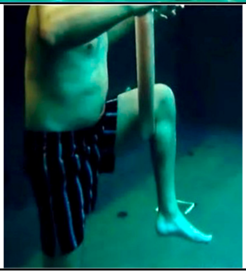
2.8. Aquatic exercise: Cycling movement	Provided injury has properly healed, the participant is in a pool with water at chest level. The participant alternately performs hip and knee extension and flexion movements simulating a pedalling movement.	**Sets:** 3<br>**Repetitions:** 10<br>**Rest between series:** 30 s	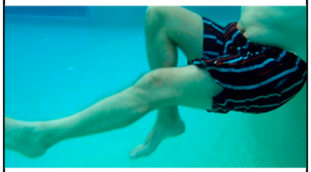
2.9. Quadriceps stretching	The subject is standing, close to a support structure only to hold on to in case of losing balance. The subject flexes the knee by bringing the heel towards the gluteal region, keeping the thigh in an upright position while avoiding pelvic anteversion by activating the gluteal and abdominal muscles. The subject slowly returns to the initial position	Maintain a tensed position for 60 s<br>**Frequency**: 3 times per week.	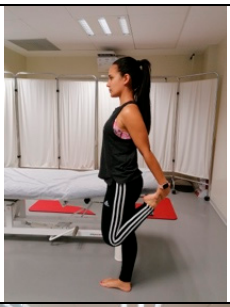
2.10. Hamstring stretching	The participant is lying supine. The healthy lower limb rests on the table. The affected lower limb is raised in hip flexion and knee extension with the help of an elastic band that the participant grasps with his hands and that rests on the anterior transverse arch of the foot. The participant stretches the knee until perceiving tension and maintains the position. Then the subject slowly returns to the initial position.	Same as exercise 2.9.	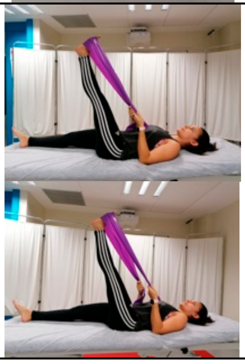

2.11. Adductor stretching	The participant is lying supine on the floor with knees straight and the pelvis as to the wall close as possible. The subject drops both legs towards abduction by sliding the heels across the wall until tension is perceived. The subject slowly returns to the initial position.	Same as exercise 2.9.	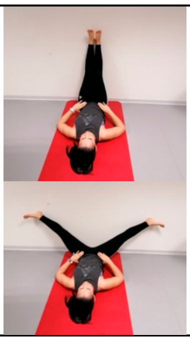
2.12. Pyramidal stretching	The participant is lying supine and flexes the hips placing the feet flat on the table. Subject then brings the foot of the affected side over the knee of the healthy side. The participant induces the stretch reflex by pushing towards hip adduction of the affected side and hip flexion of the healthy side. The tensed position is maintained. The subject slowly returns to the initial position.	Same as exercise 2.9.	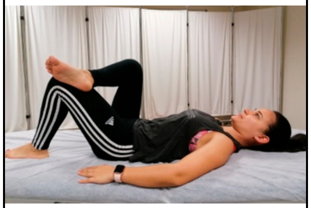
**3. MUSCLE STRENGTHENING**			
3.1. Prone active hip extension with knee extension	The participant lying prone with the head resting on the table contracts the gluteal muscles of the affected side and raises the lower limb with the knee extended towards hip extension without lifting the pelvis. Then return to the starting position.	**Sets**: 4<br>**Repetitions**: 10<br>**Sequence**: concentric 2 s, isometric 1 s, eccentric 2 s.<br>**ROM**: 0-10º hip extension<br>**Frequency**: Once a day.	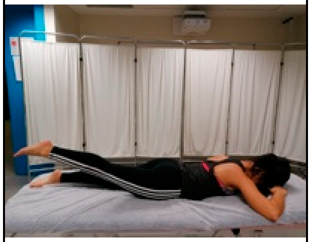
3.2. Prone active hip extension with knee in semiflexion	The subject lying prone with the head resting on the table. The participant contracts the gluteal muscles of the exercising leg and raises the lower limb towards hip extension, with the knee flexed 45º and without lifting the pelvis. Then return to the starting position.	Same as exercise 3.1.	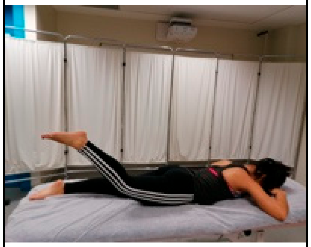
3.3. Prone active hip extension with knee flexion	The participant lying prone with the head resting on the table. The participant contracts the gluteal muscles of the exercising leg and raises the lower limb towards hip extension, with the knee flexed 90º and without lifting the pelvis. Then return to the starting position.	Same as exercise 3.1.	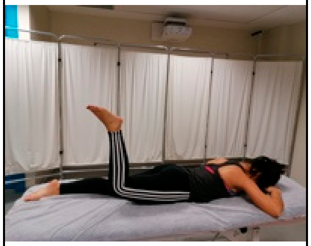
3.4. Standing active hip abduction	The participant stands in unipodal support on the healthy limb, close to a support structure to grasp only in case of loss of balance. Pelvic position must be maintained by activating the abdominal and gluteal muscles. The subject moves the affected	Same as exercise 3.1.	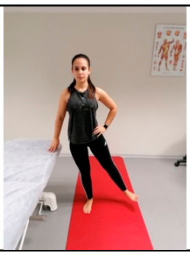

	limb away from the midline. Then return to the starting position.		
3.5. Side-lying active hip abduction	The participant is in a side-lying position with the healthy hip touching the ground; hip and knee are flexed. The arm touching the table is flexed and positioned under the patient's head. The hand and arm of the affected side rest the on the floor in front of the subject's trunk to stabilize the position. The subject raises the affected leg in the direction of hip abduction while maintaining neutral flexion-extension of the hip. Then return to the starting position.	Same as exercise 3.1.	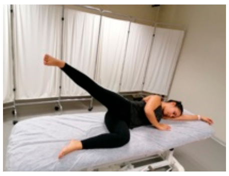 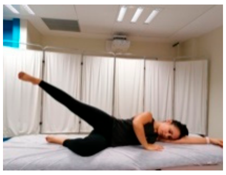
3.6. Active knee extension	The participant is in sitting position with hands resting on the table. The feet should not touch the ground. Hip and knee are flexed 90º and the lumbar spine in neutral position. The participant performs a concentric movement from 90 to 0º of knee flexion maintaining the neutral position of the pelvis. Then return to the starting position.	**Sets**: 4<br>**Repetitions**: 10<br>**Sequence**: concentric 2 s, isometric 1 s, eccentric 2 s.<br>**ROM**: 0-90º FE knee.<br>**Frequency**: Once a day.	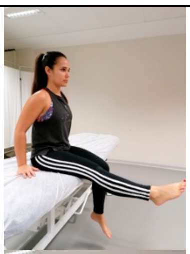 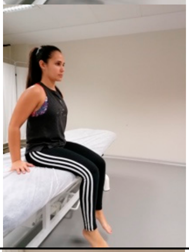
3.7. Gluteal bridge	The participant is in a supine position with the knees flexed 90º, hips flexed 45º and the feet resting flat on the table. With the hands on the abdomen, the participant lifts the pelvis until reaching the hip neutral position. Then return to the starting position.	Same as exercise 3.1.	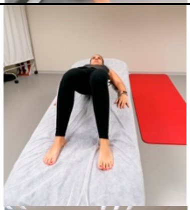 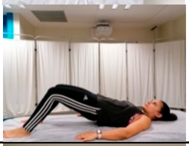
3.8. Gluteal bridge with adductor activation	The participant is in a supine position with the knees flexed 90º, hips flexed 45º and the feet resting flat on the table. A ball is placed between the knees. With the hands on the abdomen, the ball is pressed between the knees, lifting the pelvis until reaching the hip neutral position. Then return to the starting position.	Same as exercise 3.1.	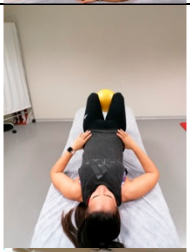 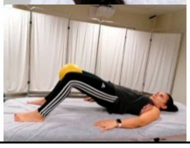

			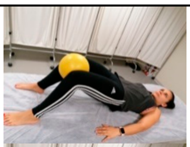 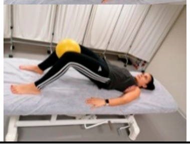
3.9. Prone resisted hip extension with knee extension	The participant is in a prone position with the head resting on the table. A 1.5-metre-long elastic band with variable resistance is placed between the ankles. The neutral position of the pelvis and hip on the healthy side must be stable. The participant contracts the gluteal muscles on the affected side and raises the lower limb towards hip extension with the knee extended against the resistance of the band. Then return to the starting position.	**Sets**: 4<br>**Repetition**: 10<br>**Sequence**: concentric 2 s, isometric 1 s, eccentric 2 s.<br>**ROM**: 0-10º hip extension<br>**Frequency**: Once a day.<br><br>**Resistance levels** (at 100% band elongation):<br>Soft (1.3 kg);<br>soft-moderate (1.7 kg);<br>moderate (2.1 kg);<br>moderate-high (2, 6 kg);<br>high (3.3 kg)	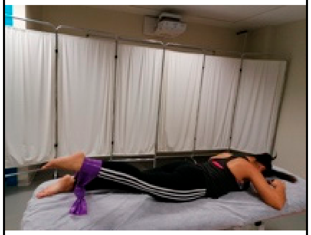
3.10. Prone resisted hip extension with knee in semiflexion	The participant is in a prone position with the head resting on the table. A 1.5-metre-long elastic band with variable resistance is placed between the ankles. The neutral position of the pelvis and hip on the healthy side must be stable. The participant contracts the gluteal muscles on the affected side and raises the lower limb towards hip extension with the knee flexed 45º against the resistance of the band. Then return to the starting position.	Same as exercise 3.9.	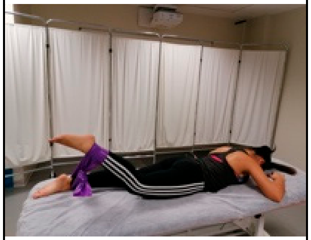
3.11. Prone resisted hip extension with knee flexion	The participant is in a prone position with the head resting on the table. A 1.5-metre-long elastic band with variable resistance is placed between the ankles. The neutral position of the pelvis and hip on the healthy side must be stable. The participant contracts the gluteal muscles of the affected side and raises the lower limb towards hip extension with the knee flexed 90º against the resistance of the band. Then return to the starting position.	Same as exercise 3. 9.	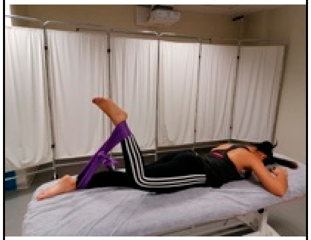

3.12. Side-lying resisted hip abduction	The subject is in a side-lying position with the healthy hip touching the floor, the hip and knee flexed. The arm touching the floor is flexed and positioned under the participant's head. The hand and arm of the affected side rests on the ground in front of the subject's trunk to stabilize the position. A 1.5-metre-long elastic band with variable resistance is placed between the ankles. The subject raises the affected leg in the direction of abduction while keeping a hip neutral flexion-extension position. Then return to the starting position.	Same as exercise 3. 9.	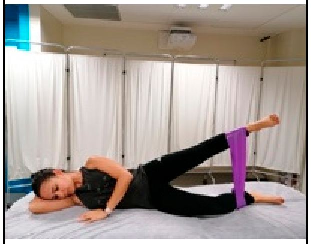
3.13. Standing resisted hip extension with knee extension	The subject is in a standing position, close to a support structure in case of loss of balance. A 1.5-metre-long elastic band with variable resistance is placed between the ankles. Pelvic position must be held neutral by activating the abdominal muscles. The subject contracts the gluteal muscles on the affected side and brings the leg back with the knee extended against the resistance of the band. Then return to the starting position.	Same as exercise 3. 9.	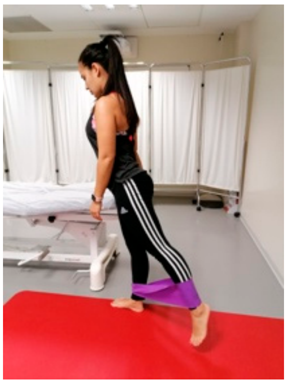
3.14. Standing resisted extension with knee in semiflexion	The subject is in a standing position, close to a support structure in case of loss of balance. A 1.5-metre-long elastic band with variable resistance is placed between the ankles. Pelvic position must be held neutral by activating the abdominal muscles. The subject contracts the gluteal musculature on the affected side and brings the leg back with the knee flexed 45º against the resistance of the band. Then return to the starting position.	Same as exercise 3. 9.	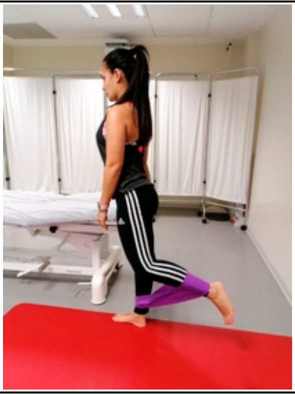
3.15. Standing resisted extension with knee flexion	The subject is in a standing position, close to a support structure in case of loss of balance. A 1.5-metre-long elastic band with variable resistance is placed between the ankles. Pelvic position must be held neutral by activating the abdominal muscles. The subject contracts the gluteal muscles on the affected side and brings the leg back with the knee flexed 90º against the resistance of the band. Then return to the starting position.	Same as exercise 3. 9.	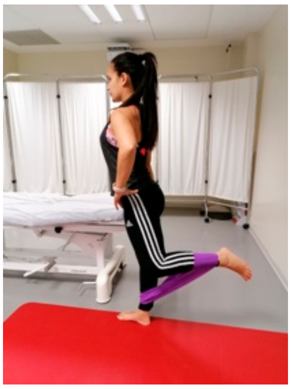

3.16. Resisted knee extension	The subject is in a sitting position with his hands resting on the stretcher. Feet should not touch the ground. Hip and knee in 90º flexion and the lumbar area in its neutral position. A 1.5-metre-long elastic band with variable resistance is placed between the ankles. Pelvic position and knee flexion on the healthy side must be stable. The subject performs knee extension against the resistance of the band. Then return to the starting position.	**Sets**: 4<br>**Repetitions**: 10<br>**Repeat sequence**: concentric 2 s, isometric 1 s, eccentric 2 s.<br>**ROM**: 0-90º FE knee.<br>**Frequency**: Once a day.<br><br>**Resistance levels** (at 100% elongation of elastic band): Soft (1.3 kg); soft-moderate (1.7 kg); moderate (2.1 kg); moderate-high (2, 6 kg); high (3.3 kg)	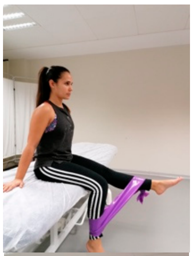 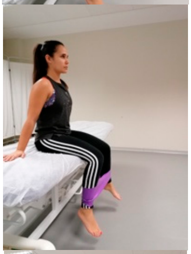 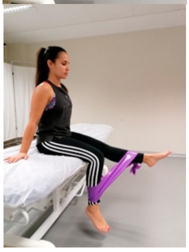
3.17. Isometric hamstring exercise with Swiss Ball	The participant lying supine with hips and knees in 90º flexion and the heels resting on a fitness ball. The subject presses heels into the ball.	**Sets**: 4<br>**Repetitions**: 10<br>**Repeat sequence**: concentric 2 s, isometric 1 s, eccentric 2 s.<br>**Frequency**: Once a day	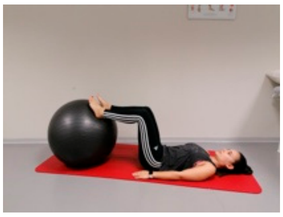
3.18. Swiss Ball wall squat	The participant is standing with a ball between his or her back and the wall. Feet should be shoulder-width apart. The subject performs knee flexion up to 40º. Then return to the starting position.	**Sets**: 4<br>**Repetitions**: 10<br>**Sequence**:<br>2 s eccentric<br>1 s isometric 2 s concentric.<br>**Rest between repetitions**: 3 s<br>**Frequency**: Once a day	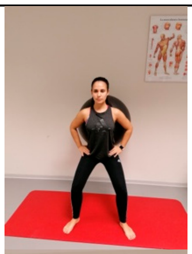 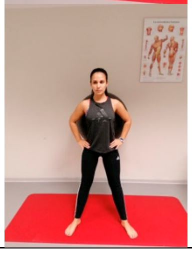

4. NEUROMUSCULAR CONTROL			
**A. PROPIOCEPTION EXERCISES**			
4.1. Load transfer	The participant is in a standing position with the feet shoulder-width apart. The subject shifts the body weight from one leg to the other holding each position for 6 s. Then return to the starting position.	**Sets**: 2<br>**Repetitions**: 10<br>Hold each position for 6 s.<br>**Frequency**: Once a day	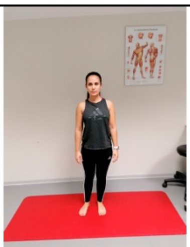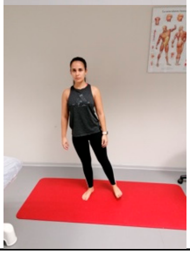
4.2. Monopodal stance with knee flexed	The participant stands with feet placed shoulder-width apart. Subject raises the healthy leg standing on the affected leg in unipodal support. The position is held for 6 s. Then return to the starting position.	**Sets**: 2<br>**Repetitions**: 10<br>6 s in monopodal support<br>**Rest between repetitions**: 6 s<br>**Frequency**: Once a day	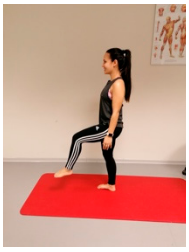
4.3. Proprioception on unstable base	The participant stands in monopodal support on an unstable base (Bosu or Dyna Disc-type), knee and hip unlocked. The participant is close to a support structure in case of loss of balance. The position is held in monopodal support. Then return to bilateral stance.	**Sets**: 1<br>**Repetitions**: 6<br>**Time each repetition**: 20 s<br>**Rest between repetitions**: 30 s<br>**Frequency**: Once a day	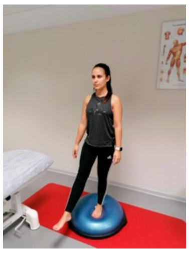

4.4. Proprioception on an unstable base with active hip movement	The participant is in monopodal support on an unstable base (Bosu or Dyna Disc-type), knee and hip unlocked. Subject is close to a support structure in case of loss of balance. The position is held in monopodal support while performing active hip abduction, adduction, flexion and extension movements. Then return to bilateral support.	Same as exercise 4.3.	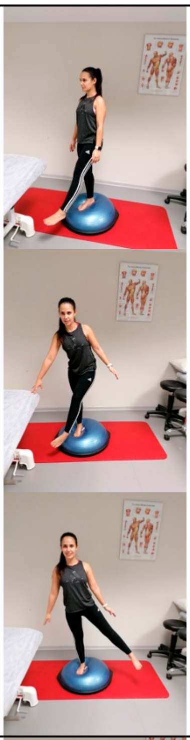
4.5. Proprioception on an unstable base with elastic band	The participant is in monopodal support on an unstable base (Bosu or Dyna Disc-type), knee and hip unlocked. A 1.5-metre-long elastic band with variable resistance is placed between the ankles. Subject is close to a support structure in case of loss of balance The position is held in monopodal support while performing active resisted hip abduction, adduction, flexion and extension. Then return to bilateral support.	Same as exercise 4.3.	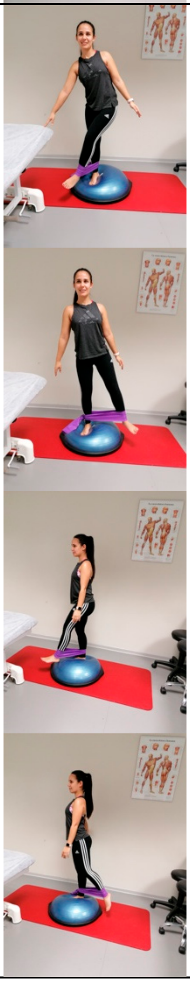

4.6. Gluteal bridge with unilateral support	The participant is lying supine, hips flexed 45º, knees flexed 90º, and feet flat on the ground. An elastic band is placed between the knees. The participant abducts the hip to shoulder-width against the resistance of the elastic band, then lifts the pelvis until the hip is in its neutral position, and completely straightens one knee. Then return to the starting position.	**Sets**: 2<br>**Repetitions**: 10<br>**Sequence**:<br>2s concentric<br>1s isometric<br>2s eccentric<br>**Rest between repetitions**: 30s	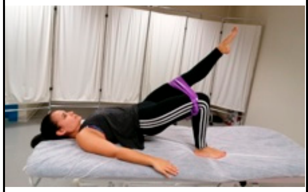
**B. WALKING**			
4.7. Walking heel to toe in water	If the injury has properly healed, the participant stands with the water at chest level. Subject slow walks normalizing the physiological heel to toe walking.	**Sets**: 4<br>**Repetitions**: 20<br>**Rest between sets**: 30s	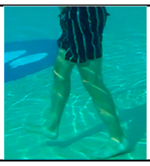
**5. CARDIOVASCULAR TRAINING**			
5.1. Stationary bicycle	The subject sits on a static bike with a raised saddle to avoid wide hip flexion. Initially without resistance and progressively increasing intensity.	15 -25 minutes.<br>**Frequency**: 3 times per week	
5.2. Elliptical movement	The subject stands on an elliptical trainer and performs low-intensity elliptical movements.	15 minutes<br>**Frequency**: 3 times per week	
**6. TRUNK EXERCISES**			
6.1. Side-lying isometrics using knees	The subject is in a side-lying position with knees and forearm against the floor. The participant lifts the pelvis until the hip is in a neutral position. Then return to the starting position.	**Sets: 1**<br>**Repetitions**: 5<br>**Time repetition**: isometric contraction increased progressively from 30s to 80s<br>**Rest between repetitions**: 30 s	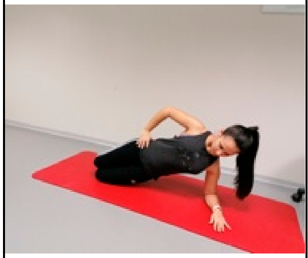
6.2. Lying-face-down isometrics using knees	The subject is lying face down with knees and forearms against the floor. The participant lifts the pelvis until the hip is in a neutral position. Then return to the starting position.	**Sets**: 1<br>**Repetitions**: 5<br>**Time repetition**: isometric contraction increased progressively from 30s to 80s<br>**Rest between repetitions**: 30 s	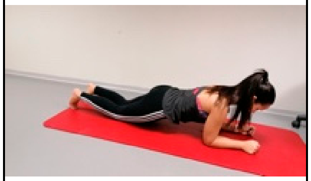
6.3. Exercise ball sit-ups	The participant sits on a gym ball, with hands behind the head. With the abdominal muscles contracted, subject takes steps forward until lying with the lumbar spine on the ball. Then return to the starting position.	**Sets**: 3<br>**Repetitions**: 5<br>**Sequence**:<br>2s concentric,<br>1s isometric,<br>2s eccentric<br>**ROM**: 90º to 0º hip flexion.	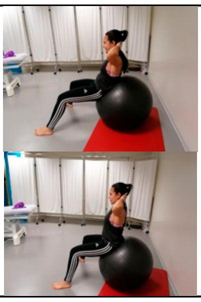

6.4. Quadruped extension	The participant in quadruped position with knees and hips vertically aligned, and hands and shoulder vertically aligned. Subject then extends contralateral lower limb and ipsilateral upper limb. Then returns to the starting position.	Same as exercise 6.3.	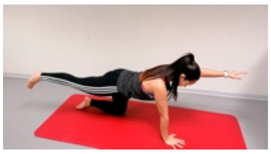

ROM, Range of motion; s, seconds.

2. Müller&Puig protocol

The main differences between the control group and the experimental group are shown in the next comparative table.

Table S3. Differences between the control group and the experimental group.

Control group	Experimental group
Global strengthening without any preferred muscle group	Strengthening of hip abductors, hip and knee extensors, as a priority, in addition to the deep hip rotators.
Analytical stretching of the lower limb muscles, without any preferred group.	Stretches of quadriceps, hamstrings and deep hip rotators begin in week 8
There is no stabilization work.	Stabilization exercises of CORE
There is no specific proprioceptive work.	Intensification of stability and proprioception work from week 6 through the use of unstable surfaces.

The control group followed a non-specific work of strengthening and stretching of all muscle groups of the lower limb, without global stabilization or proprioceptive work. The experimental group followed an activation of hip abduction and extension and knee extension, began stretching from week 8, and included CORE stabilization exercises and proprioception on unstable surfaces.

Table S4. Control protocol

EXERCISE	DESCRIPTION	INTENSITY AND FREQUENCY
**WEEKS 1-4**		
Education program	Increased rest<br>Postural education in sitting, gait, sleeping, lifting and carrying, washing and bathing.<br>Avoid cross-legged in seated position	

	Avoid static postures for extended periods.<br>Avoid combination of flexion, adduction and internal rotation of the hip.<br>Avoid full squats<br>Avoid pivoting on the affected side	
Strengthening:<br>Active hip mobility	**Flexo-extension**: In standing position over the healthy side the affected lower limb is moved forwards and backwards.<br>**Abdo-adduction**: In standing position over the healthy side the affected lower limb is moved from in frontal plane.<br>**Internal and external rotation**: In supine position roll the lower limb from inside to outside.	Sets: 3<br>Repetitions: 10<br><br>Times a week: 4
Strengthening:<br>Isometric	In standing position push hip in neutral position push the lower limb in lateral and antero-posterior plans alternatively.	Sets: 3<br>Repetitions: 10<br>6 s per contraction.<br>Rest between repetitions: 3 s.<br>Frequency: Once a day
	**WEEKS 5-8**	
Strengthening:<br>Resisted hip movements	The subject is in a standing position, close to a support structure in case of loss of balance. A 1.5-metre-long elastic band with variable resistance, progressively increased from soft to moderate, is placed between the ankles.<br>**Flexo-extension**: the participants move the lower limb in anteroposterior plane.<br>**Abdo-adduction**: the participants move the lower limb in lateral plane.<br><br>**Resistance levels** (at 100% band elongation):<br>Soft (1.3 kg);<br>soft-moderate (1.7 kg);<br>moderate (2.1 kg);	**Sets**: 4<br>**Repetition**: 10<br>**Sequence**: concentric 2 s, isometric 1 s, eccentric 2 s.<br>**Frequency**: Once a day.
Strengthening:<br>Resisted knee movemen ts	The subject is in a seating position. A 1.5-metre-long elastic band with variable resistance, progressively increased from soft to moderate is placed between the ankles.<br>**Flexo-extension**: the participants straight the affected knee in anteroposterior plane.<br><br>**Resistance levels** (at 100% band elongation):<br>Soft (1.3 kg);<br>soft-moderate (1.7 kg);<br>moderate (2.1 kg);	**Sets**: 4<br>**Repetition**: 10<br>**Sequence**: concentric 2 s, isometric 1 s, eccentric 2 s.<br>**Frequency**: Once a day.
Quadriceps stretching	The subject is standing. The subject flexes the knee by bringing the heel towards the gluteal region, keeping the thigh in an upright position while avoiding pelvic anteversion by activating the gluteal and abdominal<br>muscles. The subject slowly returns to the initial position	Maintain a tensed position for 60 s<br>**Frequency**: 3 times<br>per week.
Hamstring stretching	The participant is lying supine. The healthy lower limb rests on the table. The affected lower limb is raised in hip flexion and knee extension with the help of an elastic band that the	Maintain a tensed

	participant grasps with his hands and that rests on the anterior transverse arch of the foot. The participant stretches the knee until perceiving tension and maintains the position. Then the subject slowly returns to the initial pos ition.	position for 60 s<br>**Frequency**: 3 times per week.
Adductor stretching	The participant is lying supine on the floor with knees straight and the pelvis as to the wall close as possible. The subject drops both legs towards abduction by sliding the heels across the wall until tension is perceived. The subject slowly returns to the initial positio n.	Maintain a tensed position for 60 s<br>**Frequency**: 3 times per week.
Stretching	Flexion<br>Extension<br>Abduction<br>Aduction<br>Internal rotation<br>External rotation	
Functional activities	Walking progressively for short periods	(Every day beginning in less than 30 minutes)
**WEEKS 9-14**		
Strengthening: Resisted hip movemen ts	The subject is in a standing position, close to a support structure in case of loss of balance. A 1.5-metre-long elastic band with variable resistance, progressively increased from moderate to high, is placed between the ankles.<br>**Flexo-extension**: the participants move the lower limb in anteroposterior plane.<br>**Abdo-adduction**: the participants move the lower limb in lateral plane.<br><br>**Resistance levels** (at 100% band elongation):<br>moderate (2.1 kg);<br>moderate-high (2, 6 kg);<br>high (3.3 kg)	**Sets**: 4<br>**Repetition**: 10<br>**Sequence**: concentric 2 s, isometric 1 s, eccentric 2 s.<br>**Frequency**: Once a day.
Strengthening: Resisted knee moveme nts	The subject is in a seating position. A 1.5-metre-long elastic band with variable resistance, progressively increased from moderate to high is placed between the ankles.<br>**Flexo-extension**: the participants straight the affected knee in anteroposterior plane.<br><br>**Resistance levels** (at 100% band elongation):<br>moderate (2.1 kg);<br>moderate-high (2, 6 kg);<br>high (3.3 kg)	**Sets**: 4<br>**Repetition**: 10<br>**Sequence**: concentric 2 s, isometric 1 s, eccentric 2 s.<br>**Frequency**: Once a day.

Quadriceps stretching	The subject is standing. The subject flexes the knee by bringing the heel towards the gluteal region, keeping the thigh in an upright position while avoiding pelvic anteversion by activating the gluteal and abdominal muscles. The subject slowly returns to the initial positio n	Maintain a tensed position for 60 s **Frequency**: 3 times per week.
Hamstring stretching	The participant is lying supine. The healthy lower limb rests on the table. The affected lower limb is raised in hip flexion and knee extension with the help of an elastic band that the participant grasps with his hands and that rests on the anterior transverse arch of the foot. The participant stretches the knee until perceiving tension and maintains the position. Then, the subject slowly returns to the initial p osition.	Maintain a tensed position for 60 s **Frequency**: 3 times per week.
Adductor stretching	The participant is lying supine on the floor with knees straight and the pelvis as to the wall close as possible. The subject drops both legs towards abduction by sliding the heels across the wall until tension is perceived. The subject slowly returns to the initial positi on.	Maintain a tensed position for 60 s **Frequency**: 3 times per week.
Flexors stretching	The participant is lying supine on the table. The healthy lower limb is flexed with foot on the table. The affected lower limb is falling out of the table.	Maintain a tensed position for 60 s **Frequency**: 3 times per week.
Functional activities	Walking for progressively longer periods (even more than 30 minutes)	
